# Diagnosis of Pituitary Adenoma Biopsies by Ultrahigh Resolution Optical Coherence Tomography Using Neuronal Networks

**DOI:** 10.3389/fendo.2021.730100

**Published:** 2021-10-18

**Authors:** Alexander Micko, Fabian Placzek, Roger Fonollà, Michael Winklehner, Ryan Sentosa, Arno Krause, Greisa Vila, Romana Höftberger, Marco Andreana, Wolfgang Drexler, Rainer A. Leitgeb, Angelika Unterhuber, Stefan Wolfsberger

**Affiliations:** ^1^ Department of Neurosurgery, Medical University of Vienna, Vienna, Austria; ^2^ Center for Medical Physics and Biomedical Engineering, Medical University Vienna, Vienna, Austria; ^3^ Department of Electrical Engineering, Video Coding and Architectures, Eindhoven University of Technology, Eindhoven, Netherlands; ^4^ Division of Neuropathology and Neurochemistry, Department of Neurology, Medical University of Vienna, Vienna, Austria; ^5^ Division of Endocrinology and Metabolism of the Department of Internal Medicine III, Vienna, Austria; ^6^ Christian Doppler Laboratory Innovative Optical Imaging and its Translation for “Innovative Optical Imaging and its Translation into Medicine” (OPTRAMED), Medical University of Vienna, Vienna, Austria

**Keywords:** Optical coherence tomography, pituitary gland, pituitary adenoma (PA), transition zone (TZ), convolutional neural network (CNN)

## Abstract

**Objective:**

Despite advancements of intraoperative visualization, the difficulty to visually distinguish adenoma from adjacent pituitary gland due to textural similarities may lead to incomplete adenoma resection or impairment of pituitary function. The aim of this study was to investigate optical coherence tomography (OCT) imaging in combination with a convolutional neural network (CNN) for objectively identify pituitary adenoma tissue in an *ex vivo* setting.

**Methods:**

A prospective study was conducted to train and test a CNN algorithm to identify pituitary adenoma tissue in OCT images of adenoma and adjacent pituitary gland samples. From each sample, 500 slices of adjacent cross-sectional OCT images were used for CNN classification.

**Results:**

OCT data acquisition was feasible in 19/20 (95%) patients. The 16.000 OCT slices of 16/19 of cases were employed for creating a trained CNN algorithm (70% for training, 15% for validating the classifier). Thereafter, the classifier was tested on the paired samples of three patients (3.000 slices). The CNN correctly predicted adenoma in the 3 adenoma samples (98%, 100% and 84% respectively), and correctly predicted gland and transition zone in the 3 samples from the adjacent pituitary gland.

**Conclusion:**

Trained convolutional neural network computing has the potential for fast and objective identification of pituitary adenoma tissue in OCT images with high sensitivity *ex vivo*. However, further investigation with larger number of samples is required.

## 1 Introduction

Despite advancements of intraoperative visualization using high-definition endoscopes and image guidance, the difficulty to visually distinguish adenoma from adjacent pituitary gland due to textural similarities may lead to incomplete adenoma resection or impairment of pituitary function (1, 2).

Incomplete adenoma resection contributes to the low remission rate (47-71%) still encountered after pituitary adenoma surgery in long-term follow-up and the requirement for further surgical, medical or radiosurgical treatments (3). Inadvertently removed pituitary gland tissue can cause hormonal insufficiencies and require lifelong medical treatment. Different methods for improvement of intraoperative tissue identification have been examined like: histopathology, hormonal sampling, fluorescence agents or imaging but these techniques harbor the problem of interobserver variability or invasiveness. Therefore, novel methods for intraoperative identification of pituitary adenoma tissue have to be developed.

In the last decades, optical coherence tomography (OCT) is one of the most successfully translated imaging technique. OCT is based on low-coherence interferometry, enabling non-invasive, high resolution, three-dimensional image acquisition of investigated tissue (4). OCT has been shown a valid method for real-time differentiation of tissue microstructures in various fields of medicine such as ophthalmology (5) or urology (6). For glandular tissues, OCT has been successfully applied to detect gastrointestinal adenoma tissue *in vivo (* 7) and to intraoperatively identify parathyroid tissue (8). Further, OCT has been integrated into imaging devices such as fiber optic catheters in various medical fields, such as cardiology, gastroenterology, pulmonology, or urology (9).

We hypothesize that OCT may in future be applied for interoperative tissue interrogation during endoscopic transsphenoidal surgery to distinguish pituitary adenoma from normal pituitary gland tissue. In an initial proof-of-concept study, we co-registered OCT images with H&E slices and defined criteria for tissue differentiation: In normal pituitary gland tissue, cell nest structures and surrounding extracellular collagen matrix (that shows a particular strong signal in OCT images) were identified that allow visual distinction from adenoma samples with more than 80% accuracy (10).

The aim of the present study was to train, optimize and verify a neural networking algorithm of OCT images to identify pituitary adenoma tissue for automated tissue identification during future intraoperative application.

## 2 Methods

A prospective study was conducted to develop and test a trained convolutional neural network (CNN) algorithm to identify pituitary adenoma tissue in OCT images of adenoma and adjacent pituitary gland. Twenty patients operated for pituitary adenoma at the Department of Neurosurgery, Medical University of Vienna, Austria *via* an endoscopic approach between 2019 and 2020 were examined. This study was approved by the hospital’s ethics committee (IRB number: 1286/2018) and was done in accordance with the principles of the Declaration of Helsinki. Every gender and patient in the range of 18 to 99 years of age were included. Participants had to give their written informed consent.

Inclusion criteria were surgical cases with unequivocally MR identified pituitary adenoma and paired samples with histopathological confirmation of adenoma (according to the WHO classification of 2017) and adjacent pituitary gland tissue, respectively (11). Tissue samples with mainly necrotic tissue were excluded.

At our institution, tissue samples are routinely harvested during pituitary adenoma surgery from the central adenoma core and the border zone of the pituitary adenoma to the pituitary gland to increase remission rate. For this study, specimens were immediately snap frozen at -80°C after removal.

### 2.1 Optical Coherence Tomography

The advantage of OCT is the acquisition of three-dimensional data, enabling enface as well as cross-sectional (B-scan) tissue visualization. For OCT imaging at the Center for Medical Physics and Biomedical Engineering, Medical University of Vienna, Austria, the samples were thawed, placed on a microscope holder (D35-14-1.5N – Cellvis) and moisturized with saline solution. A modified version of a UHR-SD-OCT system was used (12, 13). This system includes a compact Ti : Sapphire laser (central wavelength at 800 nm with a bandwidth of 150 nm at the full width half maximum) operating at a power of 2.2 mW that was found not to alter the sample’s properties for histopathological examination afterwards as described in our previous publication (10).

The lateral and axial resolution was 1.4 µm and 2.9 µm, respectively. Investigators were blinded to the intraoperative sample location.

All biopsy specimen were fixed in 4% neutral buffered formalin, routinely processed, embedded in paraffin, cut in 2μm slices and stained with haematoxylin and eosin (H&E) for routine histopathological examination at the Division of Neuropathology and Neurochemistry, Vienna, Austria. The analyzing neuropathologists were blinded to the results of the OCT and intraoperative findings.

### 2.2 Convolutional Neural Network

CNNs are capable of discerning patterns in images and are highly effective for tasks such as classification or tissue segmentation. The OCT images of our cases were analyzed using CNN to distinguish pituitary gland from adenoma tissue. From each sample, 500 adjacent cross-sectional OCT images were used for CNN classification. Using the histopathological label “pituitary adenoma” or “pituitary gland”, 70% of the image data were used for training the CNN, 15% for validating the classifier and tuning the training process to optimize outcome. The remaining 15% (3 patients) were used to test the CNN to mimic automated tissue identification of new data previously unseen by the network during future intraoperative application.

### 2.3 Statistical Analysis

The data are presented as median (interquartile range) and mean (standard deviation) for continuous variables and as frequencies for categorical variables. A transfer learning approach with a pre-trained VGG16 was used for CNN in this study. The model was trained with Adam optimizer (learning rate of 1e-3 and batch size of 64) until convergence of the validation set. The results of the trained CNN were correlated with histopathological results to evaluate sensitivity to correctly identified pituitary adenoma.

## 3 Results

### 3.1 Patient and Tumor Characteristics

For this study, paired samples of 20 patients were selected, which were histopathology confirmed pituitary gland and adenoma, respectively. Median age was 55 years interquartile range (IQR) 38 -66 years, mean age 52 years, standard deviation (SD) ± 17.3. Eleven patients (55%) were female and nine patients (45%) were male. Preoperative MRI revealed a median maximal tumor diameter of 22.3 mm (IQR 12 – 28 mm; mean 21.6 mm ± 9.8), the median tumor volume was 3.8 cm^3^ (1 - 6 cm^3^; mean 5.4 cm^3^ ± 8.2). Samples of eight functioning pituitary adenomas (40%) and twelve non-functioning adenomas (60%) were included. The consistency of tumor was cystic in two tumors (10%), soft in fourteen (70%) and fibrous in four (20%) of cases. Invasiveness into surrounding structures (particularly into compartments of the cavernous sinus) was found in five cases (25%) (**Table 1**).

**Table 1:** Patient and tumor characteristics.

	Pituitary Adenoma Series	
	n (range)	%
*Patient characteristics*		
Number of patients	20	
Age	51.8 (18 – 82)	
< 65a	15	75
> 65a	5	25
Gender (female: male)	(1: 0.82)	
*Tumor characteristics*		
Functional Classification		
functional	8	40
non-functional	12	60
WHO 2017 Classification^11^		
Pit-1 plurihormonal	1	5
Lactotroph	2	10
Mammosomatotroph	3	15
Corticotroph	2	10
Gonadotroph	7	35
Null Cell	5	25
Size (max. diameter, mm)	22 (7 – 47)	
Volume (cm^3^)	5.4 (0.1 – 38)	
Consistency		
cystic	2	10
soft	14	70
fibrous	4	20
Invasiveness (direct endoscopic visualization)	5	25

### 3.2 OCT Imaging

OCT data acquisition was feasible in 19/20 (95%) patients. One biopsy pair was excluded, because in one of the two samples a previously undetected air inclusion prevented the selection of 500 adjacent slices for the CNN analysis. The acquisition time for the OCT of one sample (500 images) was approximately 5 seconds.

**Figure 1:**
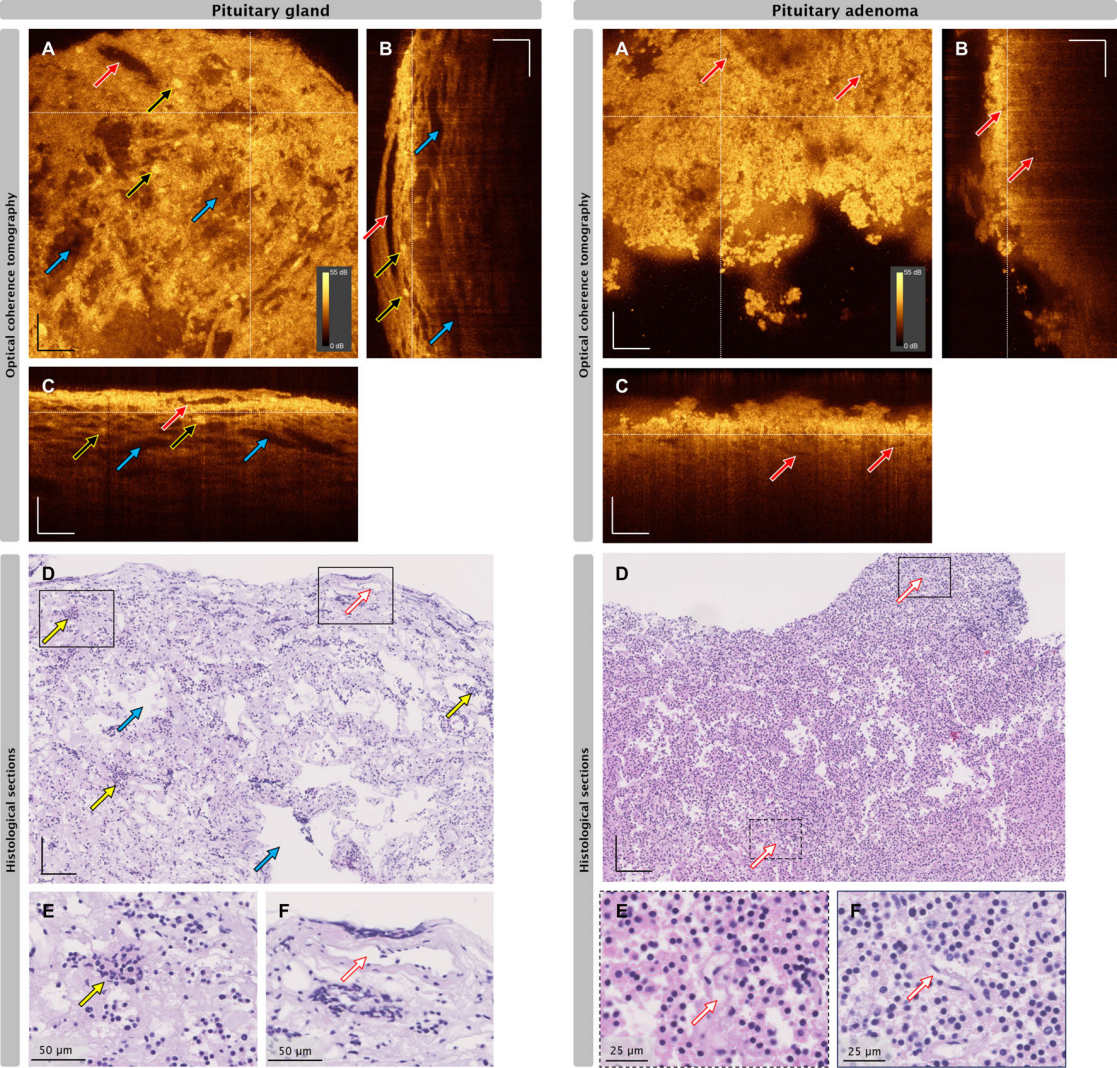
Correlation of OCT and histopathological images of pituitary gland tissue (left) and pituitary adenoma (right). **(A)** Enface OCT projections, white dotted lines indicate positions of cross-sectional OCT (B-scan) positions. **(B, C)** B-scans at the indicated positions depicted in A. **(D–F)** H&E slices of the same biopsy. Red arrows: blood vessel, yellow arrows: representative nests, blue arrows: mechanical or freezing artifacts. Scale bars without label: 100 µm. Dynamic range 0-55 dB applicable for each OCT image.

**Figure 1** depict the representative OCT images of one biopsy pair of histologically confirmed adenoma and pituitary gland, the corresponding H&E slices and cues for visual differentiation as described in our previous study.^8^ All OCT data were displayed with the same brightness and contrast settings enabling a direct comparison of signal intensities throughout the presented data.

### 3.3 CNN Classification

To quantify the differentiation capabilities of OCT, classification by CNN was performed. Therefore, 16,000 images were included in the analysis (70% for training, 15% for validating the classifier). For verification, the classifier was tested on the paired samples of three patients (3,000 images), one sample being pituitary adenoma, the other sample being adjacent pituitary gland as confirmed by histopathology.

In each case, 500 OCT slices of the adenoma and 500 OCT slices of the adjacent pituitary gland samples were examined with the trained CNN. The trained CNN could classify a single slice in less than 30ms. For a whole sample, less than 15 seconds were needed

For each slice, the CNN assigned a predictive value from 0 - 1 for the presence of adenoma tissue. To better visualize the network prediction, an average value of 150 slices was calculated.

A threshold after validation was set at a predictive value of 0.24 for maximum sensitivity to adenoma tissue. Receiver operating characteristic analysis showed an area under the curve of 0.96.

After CNN analysis of the OCT images, a detailed histopathological examination of each sample was performed.

#### 3.3.1 Case 1

44-year-old female patient presenting with frontal headache with no further clinical signs or hormonal overexpression. MRI diagnosed a 22mm diameter sellar lesion with suprasellar bulging, right parasellar extension (Knosp Grade 3A) (14) and the pituitary gland on the suprasellar surface of the suspected adenoma. Intraoperatively, fibrous tumor tissue consistency without signs of infiltration and a good cleavage plane to the pituitary gland were observed. Histopathological workup revealed a PIT-1 positive plurihormonal adenoma with expression of GH, TSH and alpha-subunit and a proliferation rate of MIB-1 of 3%. GTR and preservation of pituitary gland function were achieved.

Sample 1: Tissue from the central tumor core. CNN predicted adenoma in 98% of slices, the average prediction value was above threshold for adenoma throughout the sample. Histopathology confirmed pituitary adenoma.

**Figure 2:**
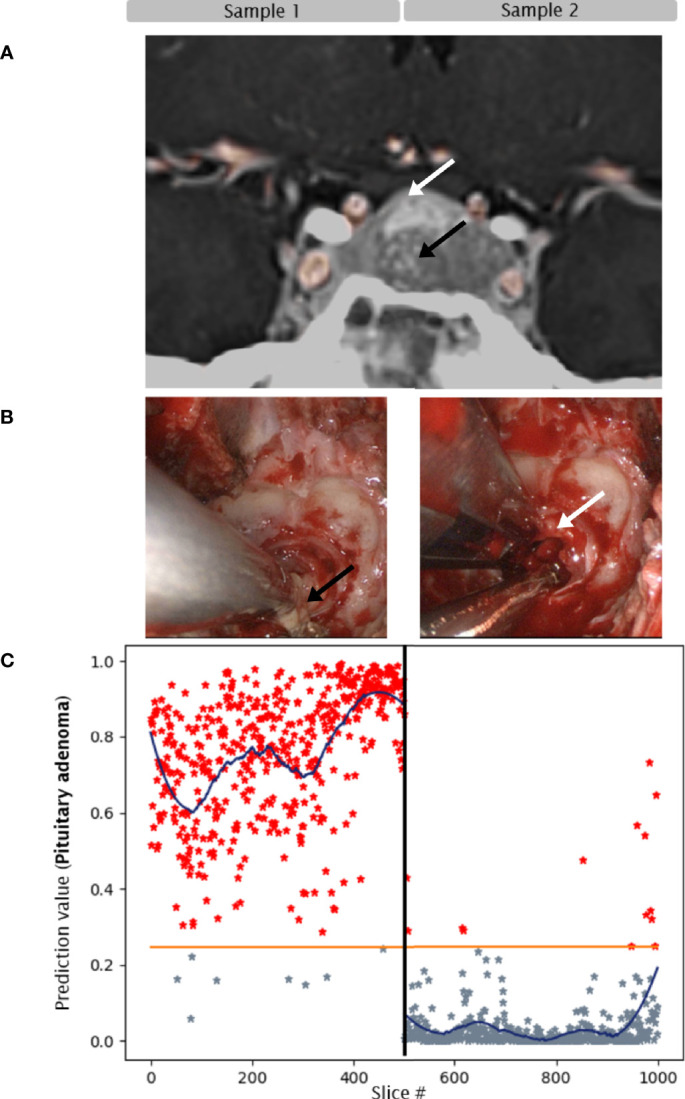
Case 1. Convolutional neuronal network assessment of OCT images of samples of pituitary adenoma (black arrow) and adjacent gland (white arrow). **(A)** MRI, 22mm macroadenoma. **(B)** intraoperative imaging. **(C)** Diagram of pituitary adenoma probability. X axis: number of each classified slice (500 per sample). The orange line indicates the classification threshold for pituitary adenoma/non-adenoma. The blue curve indicates an average of 150 slices to visualize the network prediction.

Sample 2: Biopsy from the surface of the adjacent, well separated pituitary gland. CNN predicted non-adenoma in 97% of slices, the average prediction value was below tumor threshold throughout the sample. Histopathology confirmed pituitary gland without clear-cut adenoma tissue (**Figure 2**).

#### 3.3.2 Case 2

81-year-old female patient with Cushing’s Disease (ACTH level: 99 pg/mL, normal range 7 – 64 pg/mL; 24h urinary free cortisol 264 ng/mL, normal range 4–176 ng/mL). MRI depicted a right 9mm diameter lateral endosellar microadenoma. Intraoperatively, the tumor consistency was soft without distinct pseudocapsule, hence the adjacent gland tissue was generously removed. Histopathological examination confirmed a corticotroph adenoma with a low proliferation rate of MIB-1 <1%. Endocrine remission was achieved.

Sample 1: Soft tissue from the central tumor core. CNN predicted adenoma in all slices, the average prediction value was above tumor threshold throughout the sample. Histopathology confirmed pituitary adenoma.

**Figure 3:**
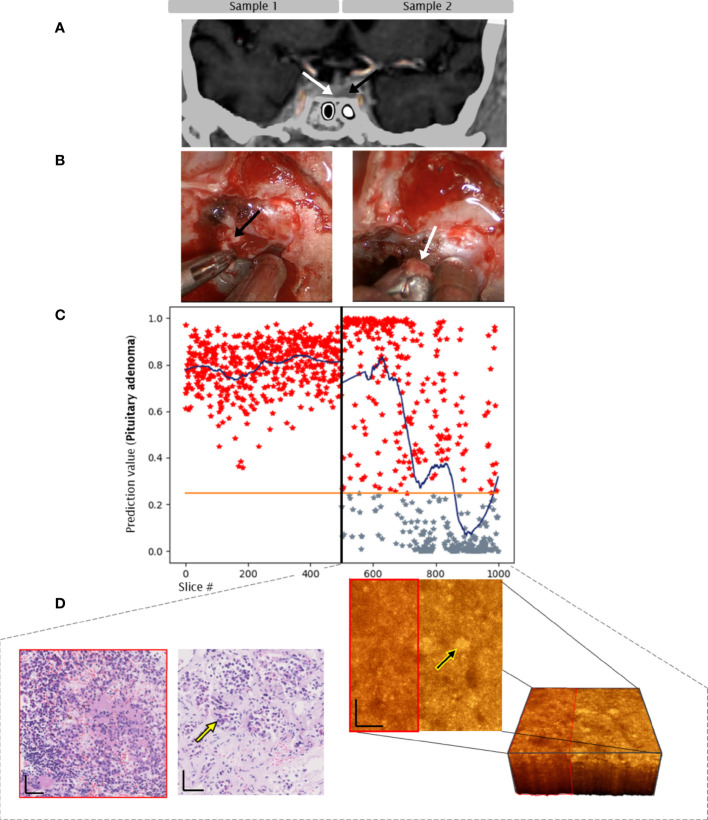
Case 2. Convolutional neuronal network assessment of OCT images of samples of pituitary adenoma (black arrow) and adjacent gland (white arrow). **(A)** MRI, 9mm microadenoma. **(B)** intraoperative imaging. **(C)** Diagram of pituitary adenoma probability. X axis: number of each classified slice (500 per sample). The orange line indicates the classification threshold for pituitary adenoma/non-adenoma. The blue curve indicates an average of 150 slices to visualize the network prediction. **(D)** OCT 3-dimensional visualization of the sub-volume of sample 2 – transition zone, H/E slices of pituitary adenoma (red box) and pituitary gland tissue. Yellow arrows indicate nests. Scale bars: 50 µm.

Sample 2: Biopsy from adjacent pituitary gland. CNN predicted adenoma tissue on one side of the sample and non-adenoma tissue on the other side of the sample. Histopathology confirmed a transition zone from adenoma to pituitary gland tissue, next to connective tissue within this section (**Figure 3**).

#### 3.3.3 Case 3

54-year-old female patient with Cushing’s Disease (ACTH level: 131 pg/mL;24h urinary free cortisol 1.147 ng/mL). MRI depicted a left lateral endosellar 8mm diameter endosellar microadenoma. Intraoperatively, the soft tumor was removed with its distinct pseudocapsule. Additionally, adjacent pituitary gland tissue was sampled. Histopathological examination confirmed a corticotroph adenoma with a high proliferation rate of MIB-1 15%. Despite GTR on MRI and initial endocrine remission, relapse of the disease occurred 6 months later. Second surgery was unsuccessful and the patient required further therapy with ketoconazole and radiation.

Sample 1: Adenoma tissue with pseudocapsule. CNN predicted adenoma in 84% of slices, from high predictive value for adenoma on one side of the sample to lower predictive value on the other side of the sample. The average prediction value remained above tumor threshold throughout the sample. Histologically, the analyzed biopsy specimen was mainly composed of fibrous tissue and contained segments of ACTH-secreting pituitary adenoma.

**Figure 4:**
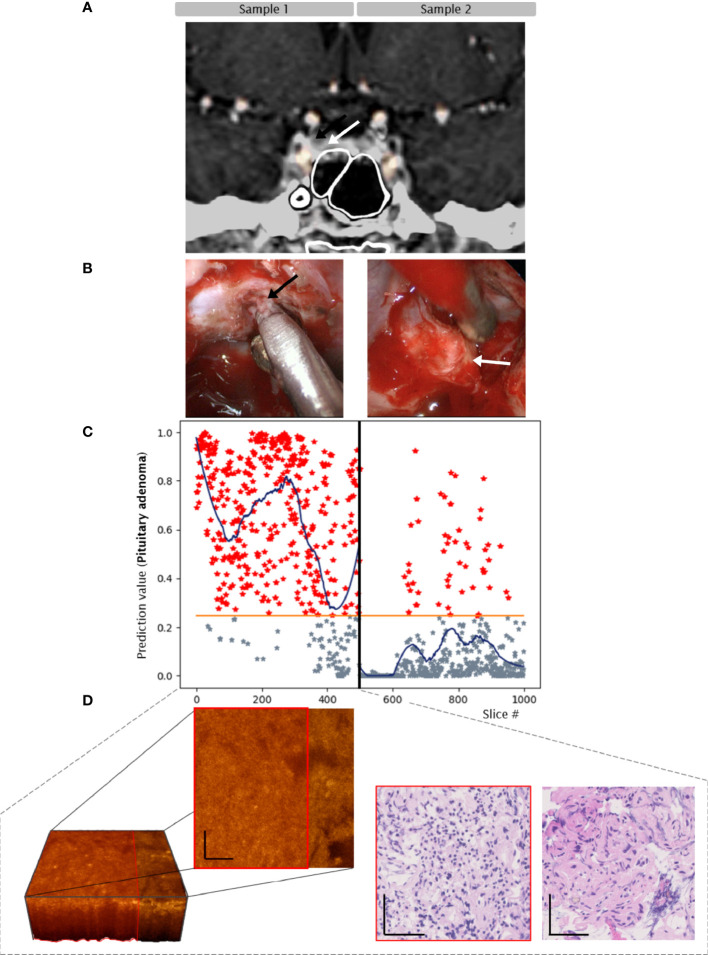
Case 3. Convolutional neuronal network assessment of OCT images of samples of pituitary adenoma (black arrow) and adjacent gland (white arrow). **(A)** MRI, 8mm microadenoma. **(B)** intraoperative imaging. **(C)** Diagram of pituitary adenoma probability. X axis: number of each classified slice (500 per sample). The orange line indicates the classification threshold for pituitary adenoma/non-adenoma. The blue curve indicates an average of 150 slices to visualize the network prediction. **(D)** OCT 3-dimensional visualization of the sub-volume of sample 1 – pituitary adenoma (red box) with connective tissue, H/E slices of segements of pituitary adenoma and fibrous tissue. Scale bars: 50 µm.

Sample 2: Biopsy from adjacent pituitary gland. CNN predicted non-adenoma in 89% of slices, the average prediction value was below tumor threshold throughout the sample. Histopathology confirmed pituitary gland without adenoma tissue (**Figure 4**).

## 4 Discussion

Differentiation of normal pituitary gland from pituitary adenoma tissue remains a surgical challenge despite improvements in visualization technology. In our present study, we found that OCT image analysis by CNN trained on 16,000 slices can predict the presence of adenoma tissue in samples from pituitary surgery with high sensitivity (84, 98 and 100%). Therefore, the intraoperative use of this technology may improve gross total resection and lower the rate of postoperative hypopituitarism in future.

During operation, the visually differentiation of pituitary adenoma and gland tissue, even with high-definition endoscopes. can be challenging (15). Therefore, various techniques have been developed to improve the correct intraoperative identification:

### 4.1 Intraoperative Histopathology

In principle, smear examinations (touch imprint) and frozen section procedures are available to intraoperatively identify tissue characteristics. For routine use to distinguish between pituitary adenoma and pituitary gland, smear sections of pituitary gland are difficult to establish due to a firmer consistency than adenoma tissue (16, 17). In frozen sections, acidophilic cells show similarities to pituitary adenoma tissue and may therefore be misinterpreted as pituitary adenoma tissue (18, 19).

Although being the most widely used techniques for intraoperative diagnostics of tumor tissue and relative inexpensive, these methods are time-consuming, harbor technical difficulties such as squashed artifacts, and despite experience are still prone to considerable interobserver variability (17).

### 4.2 Intraoperative Hormonal Sampling

To assess remaining functioning adenoma tissue, Lüdecke et al. (20) have examined intraoperative measurement of hormonal release in blood samples obtained directly from cavernous sinus compartments during different stages of pituitary surgery. The radioimmunoassays have been used for corticotroph as well as for somatotroph adenomas to check for endocrine remission (21). However, these two subtypes only represent approximately 25% of adenomas (22) and the patient has to remain for hours under general anesthesia during the hormonal sampling procedure. Although delivering a high rate of remission in cases with high baseline hormone levels, the method is invasive and harbors difficulties concerning standardization as reliable results are not present in cases with slightly elevated baseline hormone levels. Sampling of the remaining functioning pituitary adenomas as well as of non-functioning adenomas is not available to date.

### 4.3 Intraoperative Imaging

The value of visualization of remaining adenoma tissue by intraoperative MRI (iMRI) is a debated theme within the neurosurgical community. By identification of tumor remnants in up to 30% of cases after surgery of macroadenomas, it has been reported that iMRI could achieve an increase in GTR as well as ER (23–27). However, this high rate of tumor remnant detection has been criticized as potentially attributed to a cautious tumor resection keeping in mind that an iMRI will be performed (28). Although non-invasive, iMRI is time consuming, cost-intensive and therefore not widely available. Furthermore, there is a high incidence of false-positive results. Therefore, its use for resection of non-functioning adenomas has not been recommended in its latest version of the “Congress of Neurological Surgeons Systematic Review and Evidence-Based Guidelines” (29).

Various ultrasound probes adapted for transnasal transsphenoidal use have been developed in the last years which allow a high resolution 2D imaging (30). In a metanalysis by Marcus et al. (31) the intraoperative utility of ultrasound probes, which in principle could provide a relatively inexpensive and real-time imaging in comparison to other available methods mentioned before, has been investigated. In the literature they found only low-class evidence (cohort study, case series’ and case reports) reporting no safety issues but a need for advances and an experienced examiner interpreting the imaging.

### 4.4 Fluorescence Agents

To improve the rate of GTR, fluorescence agents have been preoperatively applied to visualize pituitary adenoma tissue. Five-aminolaevulinic acid, an amino acid and first compound of the porphyrin synthesis pathway, has been approved by the U.S. Food and Drug Administration in neurosurgery for intraoperatively visualization of high-grade gliomas (32–34). A compound of this pharmacologic agent accumulates predominately in tumor cells due to a lack of reduced ferrochelatase activity and becomes visible under blue light filters. However, in a recent multicenter study using a specialized blue light endoscope in none of the cases fluorescence of the pituitary adenoma tissue nor pituitary gland was found (1).

Application of indocyanine green showed equally strong fluorescence in both adenoma and pituitary gland. OLT23 a conjugate to folate receptors, has been detected by near-infrared optical imaging in a case series of 14 patients. However, this method is limited to the fact that fluorescence is dependent on an overexpression of folate receptor within the tumor which only represent a fraction of non-functioning pituitary adenoma (35).

In sum, all fluorescence agents failed to reliably distinguish all adenoma subtypes from pituitary gland tissue (36, 37).

So far, none of the techniques described above have convincingly provided a fast, non-invasive and reliable method to identify remaining adenoma tissue and pituitary gland intraoperatively.

### 4.5 Optical Coherence Tomography

By high-resolution image generation based on the scattering of photons, OCT is a fast and non-invasive technique for assessing morphological tissue information. In our previous work, we report that OCT has the potential to generate microstructural images of adenoma and gland tissue that reliably correlate with histopathology (10).

As the scale of histopathology slices is much larger than of OCT slices, the CNN analysis is more sensitive to detection of adenoma tissue e.g. in samples of transition zones that are eventually misinterpreted by histopathological analysis as gland tissue (**Case 2,** **Figure 3**). The transition zone respective pseudocapsule of the tumor is of importance, because pituitary adenomas often spread beyond the margins of the visible tumor tissue and are a source of pituitary adenoma recurrence (38).

As OCT has already been integrated into small imaging devices such as fiber optic probes, it may in future also be used during endoscopic transsphenoidal surgery to distinguish adenoma tissue from normal pituitary gland.

For such intraoperative application, fast and reliable OCT image interpretation will be required. Therefore, a CNN algorithm, trained, validated and tested with the results and data of our previous study in expansion of additional data, was investigated. Our present data verify the previous findings and moreover show, that CNN of OCT images can objectively detect pituitary adenoma tissue with high sensitivity by automatically training on the provided histopathological information per biopsy. This is the only given information for the CNN introducing a learning process to identify the best possible features to differentiate between pituitary adenoma and pituitary gland tissue.

### 4.6 Future Outlook

In patients with microadenomas, OCT may be beneficial to intraoperatively identify adenoma tissue undetected by the surgeon or invisible on preoperative MRI (e.g., in MRI-negative cases of Cushing’s Disease) to ultimately increase remission rate.

Analysis of OCT by trained CNN offers the possibility for intraoperative tissue interrogation in near real-time: The acquisition time for the OCT of the 500 images of one sample from the test cases of **Figures 2**–**4**, was approximately 5 seconds. The trained network is moreover able to classify a single B-scan in less than 30ms. The next step will be the adjustment of an existing probe for intraoperative application in endoscopic pituitary adenoma surgery. With respect to reported scanning speeds of a forward-imaging endoscopic probe, one B-scan can be acquired within 2 ms (39). The data acquisition and the classification by a trained neuronal network would be possible in less than 30 ms, which is similar to an immediate decision if adenoma tissue is present. Furthermore, inclusion of line scan Raman Microspectroscopy into the probe could deliver information about the hormonal subtype (40).

Current OCT tissue analysis in the z-direction with 800nm is approximately 0.35 mm. This limited penetration depth implies a possible limitation of gross total pituitary adenoma resection. Using longer wavelengths in the near infrared spectrum, such as 1300 nm, would lead to a deeper penetration but at the cost of contrast (39). With increased depth penetration, OCT may further be used to detect adenoma invasiveness into surrounding tissue and support the decision to remove the medial cavernous sinus wall (41).

Additionally, OCT can provide a label-free angiography contrast, which is calculated from the OCT data (42). No adjustments are necessary, despite the fact of slightly increased acquisition time: Optical coherence angiography (OCTA) was reported to be realizable with endoscopic probes and could be used to provide information of angiogenesis in greater depth (43, 44).

Inclusion of line scan Raman Microspectroscopy into the probe could deliver information about the hormonal subtype (40), but would need careful design and system integration to reject the influence of present ambient light. Adding metabolic and molecular contrast to the morphological features recorded by OCT could enable a better understanding of the underlying tissue (45), and potentially lead to an increased sensitivity in detecting adenoma cell clusters when additionally used for training a CNN.

Especially corticotroph adenomas which pose a significant challenge to the surgical team as they are often not visible on preoperative MRI, are microadenomas, show an irregular border to the surrounding normal gland tissue or even consist of islet tumor cells could be detected with an intraoperative OCT probe (46). Such a probe in addition to a trained CNN could help to identify the exact tumor borders and therefore improve remission rate and decrease the rate of postoperative hypopituitarism. However, the presence of Crooke cells in corticotroph adenomas could limit the differentiation by OCT of pituitary adenoma and pituitary gland in these tumors. Therefore, CNN training and additional information about metabolic or molecular information could help to identify tumor/gland in this kind of tumor subtypes (40, 47).

### 4.7 Limitations

A limitation of this study is the low number of cases attributed to the requirement of matching adenoma and adjacent gland tissue in each patient included.

Training is a key process for creating a neuronal network, which entirely relies on correct labeling of each slice. During histopathological workup, the sample is analyzed by a limited number of slices. If adenoma tissue is detected, the whole sample is labeled “pituitary adenoma”. This label is transferred to the CNN, which analyzes the sample at a higher resolution (500 slices) and classifies the sample in more detail. This inaccuracy ultimately reduces CNN performance because the histopathological label applied as gold standard is only correct for parts of the sample. A more precise identification of biopsy slices by manual localized histopathological labeling is a time-consuming task, but might increase the training performance of the network.

Although the trained network is already able to predict adenoma tissue with a high sensitivity with the collected dataset, a higher number of cases and more accurate data would lead to an even more robust network outcome for tissue discrimination.

A further step, bringing an endoscopic OCT system to clinical validation, would need further clinical trials or even multi-central trails. Besides approval from medical agencies, close collaboration with clinicians to develop usable systems to bridge translation of newly developed diagnostic devices from laboratories to patients bedside and implement them into clinical routine, is needed (48).

## 5 Conclusion

Trained convolutional neural network computing has the potential for fast and objective identification of pituitary adenoma tissue in OCT images with high sensitivity *ex vivo*. Due to its high tissue resolution that can identify transition zones between pituitary adenoma and gland, tissue interrogation with CNN can add value to standard histopathological analysis. However, further investigation with larger number of samples is required. Once integrated in an intraoperative probe, this novel technique may improve remission rate and preserve pituitary function during pituitary surgery in future.

## Data Availability Statement

The original contributions presented in the study are included in the article/supplementary material. Further inquiries can be directed to the corresponding author.

## Ethics Statement

The studies involving human participants were reviewed and approved by Medical University of Vienna. The patients/participants provided their written informed consent to participate in this study.

## Author Contributions

Concept and design of this study were developed by AM, FP, WD, and SW. AM, FP, and AK were responsible for data collection, AM, FP, and RF on interpretation of results and statistical analysis. MW, RH, and GV were responsible for endocrinological and neuropathological evaluation. The manuscript was prepared by AM, FP, and SW and critically reviewed by all authors. All authors contributed to the article and approved the submitted version.

## Funding

This project has received funding from the European Union’s Horizon 2020 research and innovation program under the Marie Skłodowska-Curie grant agreement No 721766 FBI, from the European Union’s Horizon 2020 research and innovation program under grant agreement No 871212 PROSCOPE and the “Medical Scientific Fund of the Mayor of the City of Vienna” and the Austrian Science Fund (DOC 33-B27).

## Conflict of Interest

The authors declare that the research was conducted in the absence of any commercial or financial relationships that could be construed as a potential conflict of interest.

## Publisher’s Note

All claims expressed in this article are solely those of the authors and do not necessarily represent those of their affiliated organizations, or those of the publisher, the editors and the reviewers. Any product that may be evaluated in this article, or claim that may be made by its manufacturer, is not guaranteed or endorsed by the publisher.
